# The Genome of *Cronobacter sakazakii* Bacteriophage vB_CsaP_GAP227 Suggests a New Genus within the *Autographivirinae*

**DOI:** 10.1128/genomeA.00122-12

**Published:** 2013-02-14

**Authors:** Reza Abbasifar, Andrew M. Kropinski, Parviz M. Sabour, Hans-Wolfgang Ackermann, Argentina Alanis Villa, Arash Abbasifar, Mansel W. Griffiths

**Affiliations:** a Canadian Research Institute for Food Safety, University of Guelph, Guelph, Ontario, Canada; b Department of Molecular and Cellular Biology, University of Guelph, Guelph, Ontario, Canada; c Public Health Agency of Canada, Laboratory for Foodborne Zoonoses, Guelph, Ontario, Canada; d Agriculture and Agri-Food Canada, Guelph Food Research Centre, Guelph, Ontario, Canada; e Department of Microbiology-Infectiology and Immunology, Faculty of Medicine, Université Laval, Quebec City, Quebec, Canada

## Abstract

The genome of *Cronobacter sakazakii* podovirus vB_CsaP_GAP227 was fully sequenced. The DNA of this lytic phage consists of 41,796 bp and has a G+C content of 55.7%. Forty-nine open reading frames and no tRNAs were identified. This phage is related to *Yersinia* phages ϕR8-01 and ϕ80-18 and *Aeromonas* phage phiAS7.

## GENOME ANNOUNCEMENT

*Cronobacter sakazakii* is a life-threatening opportunistic pathogen that causes infections in immunocompromised individuals of all ages (1). Cases of sepsis, necrotizing enterocolitis, and meningitis caused by *Cronobacter* in neonates and infants have been associated with contaminated milk-based powdered infant formulas (2, 3). As alternative agents, bacteriophages (phages) have been used to control pathogens due to their high specificity and effectiveness (4, 5). Because *Cronobacter* is antibiotic-resistant (3), phages might be useful for controlling this pathogen. However, before application, it is necessary to obtain sufficient information about the use of a particular phage as a controlling agent to guarantee its safety. Presently, only nine *Cronobacter* phages have been reported, including five myoviruses (GAP31, GAP161, ESSI-2, ES2, and CR3) (6–10), three siphoviruses (ESP2949-1, phiES15, and ENT39118) (11–13), and the unclassified phage ENT47670 (GenBank accession no. HQ201308).

Using the method described by Van Twest and Kropinski (14), *C. sakazakii* lytic phages were isolated from sewage samples (Guelph, ON, Canada). Phage vB_CsaP_GAP227 (GAP227) has a broad host range and strong lytic activity and therefore was chosen for further study. This phage lysed 10 of 14 (71.4%) *C. sakazakii* strains plus 7 of 9 (77.7%) *Cronobacter* species tested. For electron microscopy at the University of Guelph, phage preparations were negatively stained (2% uranyl acetate), and particle sizes were verified at Laval University. GAP227 has an icosahedral head of 60 nm and a tail of 10 nm, which indicates that this phage belongs to the family *Podoviridae* (15).

The Midi Lambda DNA kit (Qiagen, Mississauga, ON, Canada) was used to extract and purify the phage DNA, and determination of the genomic sequence was conducted using 454 technology (McGill University and Génome Québec Innovation Centre, Montreal, QC, Canada). By Rapid Annotations using Subsystems Technology (myRAST), the genome was annotated, and gene calls were confirmed in Kodon (Applied Maths, Austin, TX). To find the number of amino acids, the molecular weight, and the isoelectric point of each protein, Batch MW and pI Finder was used (http://greengene.uml.edu/programs/FindMW.html). To identify homologs, Batch BLAST was used (http://greengene.uml.edu/programs/NCBI_Blast.html). Transmembrane Hidden Markov Model (TMHMM) (http://www.cbs.dtu.dk/services/TMHMM-2.0/), Phobius (http://phobius.sbc.su.se/), and Pfam (http://pfam.sanger.ac.uk/) were applied to predict protein motifs.

The 41,796-bp double-stranded DNA (dsDNA) genome of phage GAP227 possesses a G+C content of 55.7% and contains 49 open reading frames (ORFs). Discontiguous megablast analysis (16) revealed a significant sequence similarity to *Yersinia* phages ϕR8-01 (GenBank accession no. HE956707) and ϕ80-18 (GenBank accession no. HE956710) and to *Aeromonas* phage phiAS7 (17). CoreGenes (18) comparative proteomic analyses confirmed these results, showing that GAP227 shared 71.4% of proteins with ϕR8-01 and ϕ80-18 and 63.3% with phiAS7. Since these values are considerably higher than those for shared proteins with the type virus ϕKMV (36.7%) (19), we propose that these four phages should be grouped in a new genus within the subfamily *Autographivirinae*.

The proteome of phage GAP227 was screened using BTXpred server to detect potential bacterial toxins (20). Since none were detected, phage GAP227 can be used as a potential controlling agent against *C. sakazakii*.

### Nucleotide sequence accession number.

The complete genome sequence of phage vB_CsaP_GAP227 is available in GenBank under the accession no. KC107834.
